# Advances in Heterocatalysis by Nanomaterials

**DOI:** 10.3390/nano10040609

**Published:** 2020-03-26

**Authors:** Ioannis V. Yentekakis, Wei Chu

**Affiliations:** 1 Physical Chemistry & Chemical Processes Laboratory, School of Environmental Engineering, Technical University of Crete (TUC), 73100-Chania, Crete, Greece; 2 Key Laboratory of Green Chemistry and Technology of Ministry of Education (MOE), College of Chemical Engineering, Sichuan University, Sichuan 610065, China

## Overview

Heterogeneous catalysis played, plays, and will continue to play a major key role in industrial processes for the large-scale synthesis of commodity chemicals of global importance [1,2] and in catalytic systems that possess a critical role in energy generation [3,4,5,6,7,8] and environmental protection approaches [9,10,11,12,13]. As a result of the ongoing progress in materials science, nanotechnology, and characterization methods, great advances have been currently recorded in heterogeneous catalysis by nanomaterials, the so-called “nanocatalysis” [14,15,16]. Efficient approaches and advanced methods for the design of nanostructured composite materials (up to atomic level) [14,15,16,17,18], subject to specific nanomorphologies with enhanced metal–metal and metal–support interactions that are favorable for catalysis (that enable fine-tuning of the critical physicochemical properties of the designed catalysts) [18], provide optimized catalytic systems with outstanding performances in numerous eco-friendly and cost-effective applications. Under this line, great progress has been achieved in many applications of heterogeneous catalysis involving, for example, emissions control catalysis, waste treatment, photocatalytic, biorefinery, CO_2_ utilization, and fuel cells applications, as well as hydrocarbon processing for H_2_, added-value chemicals, and liquid fuels production, among several others [1,2,3,4,5,6,7,8,9,10,11,12,13,14,15,16,17,18]. 

## This Special Issue: Contributions and Highlights

In this context, this Special Issue of Nanomaterials has succeeded in collecting 10 high-quality contributions that cover recent research progress in the field, involving rational synthesis and adequate characterization of novel nanostructured catalysts with improved efficiency and performance in several high-impact environmental, energy, added-value chemicals/organics synthesis and biotransformation processes, including: (i) the synthesis of added-value chemicals/organics and biotransformations (5 papers), (ii) photocatalytic pollutants degradation (2 papers), (iii) photocatalytic or electrocatalytic water splitting for H_2_ and/or O_2_ evolution (2 papers), and (iv) wastewater cleaning from pharmaceuticals (1 paper). The apparent dispersion of the application subjects and targets of these 10 paper contributions declares the prospect and importance of nanomaterials in all the directions of the area of heterogeneous catalysis.

### (i) Nanomaterials for Chemicals/Organics Synthesis and Biotransformation Applications

Su et al. [19] synthesized Rh nanoparticle catalysts dispersed on TiO_2_ and boron-decorated TiO_2_ nanotube supports (Rh/TNTs, Rh/B-TNTs), which were evaluated on the hydroformylation of 2-methyl-3-butennitrile (2M3BN). Given that 2M3BN can be used for the preparation of adiponitrile after isomerization, and the hydroformylation of 2M3BN is an industrially important and scientific research subject, in addition to the fact that hydroformylation processes are related with difficulties upon recovery of the homogeneous catalysts employed in industrial practice, the present study, employing heterogeneous catalysts, is of considerable interest. A variety of techniques, namely X-ray diffraction (XRD), scanning electron microscopy (SEM), transmission electron microscopy (TEM), X-ray photoelectron spectroscopy (XPS), temperature-programmed desorption of ammonia (NH_3_-TPD), inductively coupled plasma–atomic emission spectroscopy (ICP-AES), and Brunauer–Emmett–Teller (BET) were used for the characterization of catalysts. Superior olefin hydroformylation catalytic performance (activity, selectivity to aldehydes) of Rh/B–TNTs in comparison to Rh/TNTs was obtained, resulting from its improved acidity. A better dispersion of Rh nanoparticles on Rh/B-TNTs than that on Rh/TNTs was achieved with average particle sizes of 2.8 and 4.9 nm, respectively, while B-modification of the support also improved catalytic stability.

Riyadh et al. [20] synthesized chitosan (a natural polysaccharide)–MgO hybrid nanocomposite by a simple, one-pot precipitation method, which was characterized by means of Fourier transform pectroscopy (FTIR), elemental analysis (EDX), and scanning electron microscopy (SEM). This chitosan nanocomposite is a three-dimensional, cross-linked, polymeric matrix of chitosan with active NH and OH functional groups, incorporating MgO nanoparticles, and it can be used as a novel basic heterogeneous catalyst in the form of a solid film. The material served here as a powerful eco-friendly basic catalyst under microwave irradiation in the synthesis of two novel series of 5-arylazo-2-hydrazonothiazoles 4a–j and 2-hydrazono [1,3,4] thiadiazoles 8a–d, incorporating a sulfonamide group. Its catalytic performance, as a green recyclable catalyst, was comparatively evaluated by means of triethylamine (a traditional catalyst). The significantly better yields of the chitosan-MgO toward hydrazonothiazoles and hydrazono [1,3,4] thiadiazoles was attributed to the obtained nanosized MgO and the synergistic effect that is created by the combination of the basic nature of both MgO and chitosan. The novel nanocomposite catalyst can be easily recovered and reused for many times without loss in its catalytic activity, therefore making it promising for implementation in many other organic transformations.

Zhao et al. [21] produced ZrO_2_ nanoparticles, ZrO_2_(P) and ZrO_2_(H), with different tetragonal phase content (higher in the former), which was used as support for the preparation of 10 wt % Ni/ZrO_2_ catalysts via impregnation. The catalysts were characterized by means of XRD, Raman, H_2_-TPR, XPS, and H_2_-TPD techniques, and their catalytic performance was evaluated under the hydrogenation of maleic anhydride toward succinic anhydride and γ-butyrolacetone. The Ni/ZrO_2_(P) catalyst exhibited stronger metal–support interactions than the Ni/ZrO_2_(H) due to its higher number of oxygen vacancies and the low-coordinated oxygen ions on its surface, resulting in smaller Ni particles and higher C=C hydrogenation activity for maleic anhydride to succinic anhydride. However, the C=O hydrogenation activity of Ni/ZrO_2_(P) was found to be much lower than that of the Ni/ZrO_2_(H). A 43.5% γ-butyrolacetone yield over the Ni/ZrO_2_(H) versus a much lower of only 2.8% over the Ni/ZrO_2_(P) catalyst, at 210 °C and 5 MPa of H_2_ pressure, was obtained. In situ FTIR characterization revealed that the high C=O hydrogenation activity for the Ni/ZrO_2_(H) could be attributed to the surface synergy between active metallic nickel species and relatively electron-deficient oxygen vacancies. The obtained insight could stimulate new strategies for ZrO_2_-based catalysts performance optimization under α, β-unsaturated aldehyde, and ketone hydrogenation reactions by modulating the surface structure of ZrO_2_ supports.

Wu et al. [22] investigated a series of zeolites with different topology structures, including SAPO-34, SUZ-4, ZSM-5, USY, MOR, and beta, as catalysts for the synthesis of polyoxymethylene dimethyl ethers (PODEn) from dimethoxymethane (DMM) and trioxymethylene (TOM). Both experimental and theoretical studies were employed to evaluate the influence of acidic properties and textural/morphological characteristics of the zeolites on their activity. It was confirmed that a pore mouth diameter larger than a TOM molecule was an essential prerequisite for the synthesis of PODEn over zeolites; the synergistic effect between medium-strong Brønsted acid sites (Brønsted MAS) and the maximal available space of zeolites determines the catalytic performance of all zeolites studied. Mechanistic implications involve first the decomposition of DMM and TOM into methoxymethoxy groups (MMZ) and CH_2_O monomer over Brønsted MAS. Then, the steric constraint of the maximum included sphere, with an appropriate size in zeolite channels, can promote the combination of CH_2_O and MMZ to form transition species ZO(CH_2_O)*_n_*CH_3_, which reacted with the methyl-end group to form PODEn over Brønsted MAS. The reaction temperature appeared to affect product distribution (selectivity) due to changes in the activity of intermediate species, which also strongly depends on the maximum available space in zeolite channels. Overall, it is concluded that a pore mouth diameter larger than the TOM molecule, a proper amount of Brønsted MAS, and an appropriate maximum including the sphere size are necessary conditions to obtain high PODEn selectivity at low temperatures.

Gournis, Stamatis, and co-workers [23] synthesized porous carbon cuboids (PCC) and functionalized PCCox in order to develop novel β-glucosidase-based nanobiocatalysts for the bioconversion of oleuropein to hydroxytyrosol. Using non-covalent or covalent immobilization approaches, β-glucosidases from almonds and thermotoga maritima were attached for the first time on oxidized and non-oxidized porous carbon cuboids (PCC). A variety of characterization methods including Fourier transform infrared spectroscopy (FTIR), X-ray photoelectron spectroscopy (XPS), and atomic force microscopy (AFM) were employed for the adequate characterization of the bionanoconjugates. The oxidation state of the oxidized PCCs as a type of nano-support and the immobilization procedure appeared to play a key role on the immobilization efficiency or the catalytic activity of the immobilized β-glucosidases. The novel bionanoconjugates nanobiocatalysts formed efficiently catalyzed the hydrolysis of oleuropein, leading to the formation of its bioactivderivative, hydroxytyrosol, which is a phenolic compound with numerous health benefits. The bionanoconjugates exhibited high thermal and operational stability, up to 240 h of repeated use, pointing out their great prospect in various biotransformations.

### (ii) Nanomaterials for Photocatalytic Pollutants Degradation Applications

Long, Yan, and co-workers [24] in an attempt to meet the urgent need for advanced photocatalytic materials, fabricated novel visible light-driven heterostructured g-C_3_N_4_/TiO_2_ (CNT) composites, using graphitic carbon nitride (g-C_3_N_4_) as precursor and fibrous TiO_2_ via the electrospinning preparation method. The photocatalytic performance of CNT was evaluated under the rhodamine B degradation and was found to be superior to that of the commercial TiO_2_ (P25®) and electrospun TiO_2_ nanofibers. The specific CNT heterostructure and its enlarged specific surface area enhanced the photocatalytic performance, suppressing the recombination rate of photogenerated carriers while broadening the absorption range of light spectrum. Heterostructured CNTs with an appropriate proportion can rationally use visible light and significantly promote the photogenerated charges transferred at the contact interface between g-C_3_N_4_ and TiO_2_, rendering them as prospective candidates in photocatalytic pollutants degradation processes, as the authors conclude.

Under the same purpose, Bresolin et al. [25], based on its ability to absorb visible light, synthesized a methylammonium lead iodide perovskite (MAIPb), which was evaluated as a visible-light photocatalyst for the degradation of two model pollutants, rhodamine B (RhB) and methylene blue (MB). An approximately 65% photodegradation of RhB was achieved after 180 min of treatment, while the efficiency was enhanced up to 100% by assisting the process with a small amount of H_2_O_2_. The visible-light activity of the MAIPb perovskitic-structure photocatalyst was attributed to its outstanding optoelectronic properties, i.e., its ability to absorb light as well as to enhance the separation of photogenerated carriers.

### (iii) Nanomaterials for Photocatalytic or Electrocatalytic Water-Splitting Applications

Zeng and coworkers [26] under the view of using photocatalysis as a green technique to convert solar energy to chemical energy, specifically H_2_ production from water splitting, prepared ZnO and ZnO-red phosphorus heterostructures (ZnO/RP), through a facile calcination method for the first time. The materials studied in respect to their photocatalytic activity for H_2_ evolution through water splitting offered considerable efficiency and excellent photostability under AM1.5 light irradiation. The w/w ratio of RP in the ZnO/RP heterostructure was used for material optimization. It was found that ZnO/PR heterostuctures exhibit up to 20.8-fold enhancement of H_2_ production compared to bare ZnO, and moreover overcome the photocorrosion sensitivity of ZnO. This privileged water-splitting activity and stability of ZnO/PR was considered to result from the rapid transfer and effective separation of photogenerated electrons and holes between the heterointerface of ZnO and RP and the inhibited charge carrier recombination on the surface.

Wen et al. [27] recognized the fact that the oxygen evolution reaction (OER), in other words the water-splitting reaction, is a pivotal step for many sustainable energy technologies, and in order to overcome common unfavorable sluggish kinetics and high overpotentials during OER, synthesized a hybrid electrocatalyst (CoNi-ZIF-67@Ti_3_C_2_T*_x_*), i.e., an MXene supported CoNi-ZIF-67 hybrid. This was achieved by the in situ growth of bimetallic CoNi-ZIF-67 rhombic dodecahedrons on the Ti_3_C_2_T_x_ matrix via coprecipitation. They found that the inclusion of the MXene matrix produces smaller CoNi-ZIF-67 particles and increases the average oxidation of Co/Ni elements endowing the CoNi-ZIF-67@Ti_3_C_2_T_x_ with a pronounced OER electrocatalytic performance, which is much better than the IrO_2_ electocatalysts and the pure CoNi-ZIF-67. Therefore, this work shows new strategies for the development of efficient non-precious metal electrocatalysts for OER. 

### (iv) Nanomaterials for Adsorption-Based Wastewater Cleaning from Pharmaceuticals

Yan and co-workers [28] worked on a subject of growing environmental attention, that is the removal of pharmaceuticals from wastewater, in particular antibiotics, which are stable, difficult to degrade, and are able to generate antibiotic-resistant genes in microorganisms with concomitant adverse effects in ecosystems. In this context, the authors prepared magnetic *N*-doped porous carbon (MNPC) via the self-catalytic pyrolysis of bimetallic metal–organic frameworks (MOFs) and studied its efficiency on antibiotics adsorption. The as-produced material showed favorable features (e.g., high surface area and pore volume, good graphitization degree, rich N-doping, and magnetic properties), allowing it to be an endowed antibiotic adsorbent. This was experimentally revealed by testing the adsorption capacity of MNPC on norfloxacin (NOR) adsorption, which was found to be significant. A detailed experimental parametric study enabled the authors to conclude that the adsorption mechanism is mainly influenced by pore filling, electrostatic interaction, and the H-bond.
